# Subgroup analysis of scientific performance in the field of arthroplasty

**DOI:** 10.3389/fsurg.2023.1187223

**Published:** 2023-06-12

**Authors:** Milan Anton Wolf, Lars Goebel, Philipp Winter, Stefan Landgraeber, Patrick Orth

**Affiliations:** Department of Orthopaedic Surgery, Saarland University Medical Center, Homburg, Germany

**Keywords:** arthroplasty, research patterns, subgroup analysis, bibliometric, joint replacement

## Abstract

**Introduction:**

Arthroplasty is the final treatment option for maintaining mobility and quality of life in many primary degenerative and (post-) traumatic joint diseases. Identification of research output and potential deficits for specific subspecialties may be an important measure to achieve long-term improvement of patient care in this field.

**Methods:**

Using specific search terms and Boolean operators, all studies published since 1945 to the subgroups of arthroplasty listed in the Web of Science Core Collection were included. All identified publications were analysed according to bibliometric standards, and comparative conclusions were drawn regarding the scientific merit of each subgroup.

**Results:**

Most publications investigated the subgroups of septic surgery and materials followed by approach, navigation, aseptic loosening, robotic and enhanced recovery after surgery (ERAS). In the last 5 years, research in the fields of robotic and ERAS achieved the highest relative increase in publications In contrast, research on aseptic loosening has continued to lose interest over the last 5 years. Publications on robotics and materials received the most funding on average while those on aseptic loosening received the least. Most publications originated from USA, Germany, and England, except for research on ERAS in which Denmark stood out. Relatively, publications on aseptic loosening received the most citations, whereas the absolute scientific interest was highest for the topic infection.

**Discussion:**

In this bibliometric subgroup analysis, the primary scientific outputs focused on septic complications and materials research in the field of arthroplasty. With decreasing publication output and the least financial support, intensification of research on aseptic loosening is urgently recommended.

## Introduction

Arthroplasty is the final treatment option for maintaining mobility and quality of life for patients with many primary degenerative and (post-) traumatic joint diseases. The steadily increasing number of primary and revision surgeries is based on continuous scientific advancements in this field (1). Minimally invasive approaches allow for a faster rehabilitation (2), while the implementation of navigation and robotic-assisted surgery has shown potential to increase the accuracy of component placement (3–5). Moreover, ongoing research on materials and antibiotics has allowed improved treatments orf periprosthetic infections and enhanced durability of prosthetic implants (6, 7).

The number of joint replacements worldwide is expected to continuously increase in the forthcoming years (8–11). Furthermore, joint reconstruction that was once reserved for the low-demand population has been increasingly used in younger cohorts (12, 13). Even though the effect of age on implant survival has not yet been finally elucidated (12, 14), an increasing number of revision procedures can be expected in the future (15). In order to cope with the increased demand, a systematic pursuance of research and innovation is imperative.

Within the framework of a bibliometric study, the development of the scientific performance in individual research areas can be investigated in accordance with scientific standards. This involves recording all publications in a thematic field. Subsequently, by analysing the baseline data, conclusions are drawn about the quantity of published research papers. Further analyses allow conclusions on the quality of the research (e.g., impact factor of the journals, citation rate, source of funding, h-index). This enables scientifically substantiated statements and comparisons to be made on the individual areas of research. Whereby the different research areas within arthroplasty related research should receive balanced scientific attention depending on the clinical relevance. Since only a balanced research effort can lead to complete coverage of all clinical questions.

This study is based on the hypothesis that increasing publication pressure (16–19), differences in funding (20), citations and achievable impact factors (21, 22) may lead to a bias in research-/publication performance in the individual subgroups and aims to answer the following questions: (1) are there differences in publication performance between different topic areas in the field of arthroplasty? (2) Are there differences in scientific impact between the subgroups as measured by citations and impact factor? (3) Is there a difference in funding behavior between different topic areas?

## Methods and material

### Database and search strategy

The data collection was carried out using the Web of Science Core Collection of the worldwide established multi-disciplinary search platform for bibliographic database Web of Science™ (WoS) (23–26).

To include as many publications on the subject of arthroplasty as possible, the search terms arthroplast*, prostheti*, periprosth*, replacement* were combined with the joint specific searchterms: hip, shoulder, joint*, elbow, knee, ankle, femo*, humer*, tibia*, glenoid*.

The research on arthroplasty was divided by the authors into the most clinically as well as scientifically relevant subgroups: Periprosthetic infections and septic surgery (infection), aseptic loosening, surgical approach (approach), enhanced recovery after surgery (ERAS), perioperative navigation and robotic assisted surgery (navigation/robotic), and material science (materials). The most clinically and scientifically relevant keywords were used as search terms. The search terms, which can be obtained from Table 1, were further oriented to existing ones from previous bibliometric studies (27–29). Included were publications between 1945 and 2021.

**Table 1:** Representation of the subgroup specific search terms.

Infection	Aseptic loosening	Approach	ERAS	Navigation/robotic	Materials
Septic*	NOT sept*	Approach	Enhanced recovery	Computer * Assisted	Metal*
Bacteria*	NOT infect*		Fast track	Robo*	Ceramic*
Contamination	NOT Bacteria*		Short stay	Image based	Polyethyl*
Infect*	Failure		Fast recovery	Mako	Titaniu*
	Radioluc*		Rapid recovery	ROSA	Material*
	Loosen*		Eras	Navio	Wear
				Omnibiotics	Corrosion
				Navigat*	Tribolog*
					Stem
					Abrasion
					Abrasive
					Attrition
					Bearing

The boolean operator NOT excludes the respective term for the search. The boolean operator * allows various endings of the search term.

### Analysis

We utilized the analysis function of the Web of Science to identify the total number of publications, publications by individual countries, institutes, and authors, as well as their respective publication counts. Additionally, we identified funding agencies associated with the publications and the journals in which they were published. The resulting data set was then transferred to an Excel table (Microsoft Corporation, Redmond, WA). To determine relative publishing performance, further statistical processing was performed using GraphPad PRISM v. 9.3.1. (Graphpad Software, Inc, San Diego, CA).

To further investigate the funding agencies, they were manually assigned to either the private sector (industrial funding) or the non-private sector [governmental, non-profit organizations, and non-governmental organizations (NGOs)] according to their economic background which was determined by a manually online investigation.

The journals were manually ranked regarding their current impact factor (IF) of the Journal Citation Report (https://jcr.clarivate.com). For journals that are no longer published, the IF of the last year of publication was used. If the journal had been renamed in the meantime, the IF of the current journal was used. The geographical assignment was made according to the information in the Journal Citation Report.

## Results

### Publications

**Figure 1:**
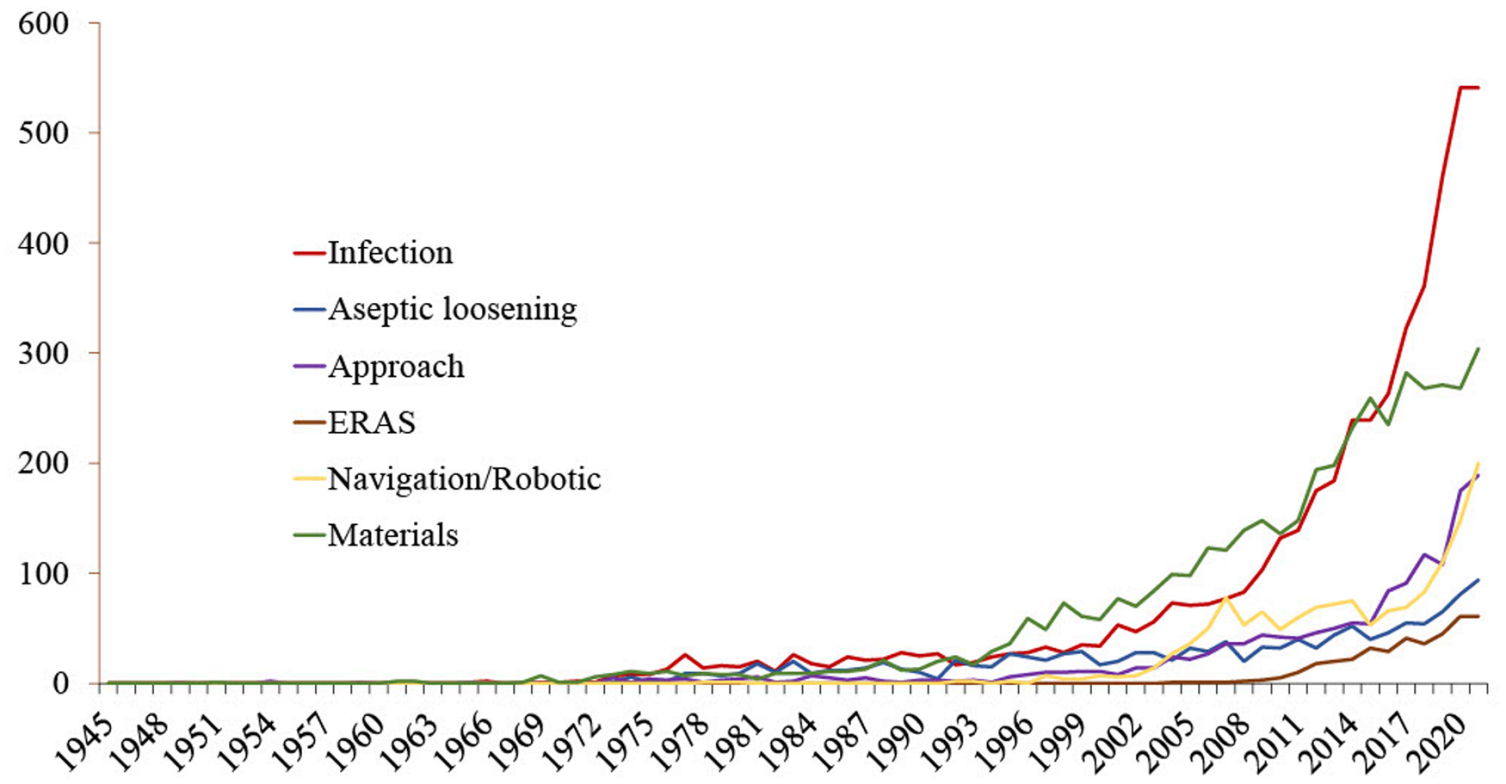
Plot of subgroup publications over time per year.

During the period under investigation, infection (4,894 publications) followed by materials (4,398 publications), navigation/robotics (1,440 publications), approach (1,422 publications), aseptic loosening (1,273 publications) and ERAS (390 publications) accounted for the largest number of publications (Figure 1).

Publications regarding materials dominated the research landscape until 2015. Since 2016, there has been an exponential increase in publications on the topic of infection (publications from 1945 to 2015: materials: 2,756, infections: 2,341; publications since 2016: materials: 1,642, infections: 2,553).

### Authors and institutes

The topics of infection and materials each had the highest number of authors and institutes (infection: 14,091 authors, 3,289 institutes; materials: 11,187 authors, 2,982 institutes). The topic ERAS had the lowest number of authors and institutes (approach: 4,998 authors, 1,587 institutes; aseptic loosening: 4,470 authors, 1,315 institutes; navigation/robotic: 4,212 authors, 1,442 institutes; ERAS: 1,297 authors, 512 institutes).

On average, more than 2 institutes participated in a publication, with the most collaborations for the ERAS topic area (institutes/publications: infection: 2.61; aseptic loosening: 2.38; approach: 2.25; ERAS: 2.82; navigation/robotic: 2.36; materials: 2.37). Most co-authorships (sum of publications of all authors /publications) were found on the topic of infection (infection: 5.41; aseptic loosening: 4.72; approach: 4.71, ERAS: 5.10; navigation/robotic: 5.03; materials: 4.78).

### Journals

A total of 1,065 different journals were used to publish on the various topics. Among these, the distribution was most diversified in absolute terms for infection and in relative terms for ERAS (infection: 565 journals, 0.12 journals/publication, aseptic loosening: 244 journals, 0.19 journals/publication; approach: 283 journals, 0.20 journals/publication; ERAS: 122 journals, 0.31 journals/publication; navigation/robotic: 187 journals, 0.13 journals/publication; materials: 465 journals, 0.11 journals/publication).

The average impact factor of the journals in which research papers were published was highest for materials and lowest for navigation/robotic (infection: 4.17, aseptic loosening: 4.16; approach: 3.24; ERAS: 3.97; navigation/robotic: 3.06; materials: 4.54).

### Funding

In research on the topic of materials, publications received the highest average funding (infection: 0.5 fundings/publication; aseptic loosening: 0.33 fundings/publication; approach: 0.32 fundings/publication; ERAS: 0.46 fundings/publication; navigation/robotic: 0.36 fundings/publication; materials: 0.57 fundings/publication).

**Figure 2:**
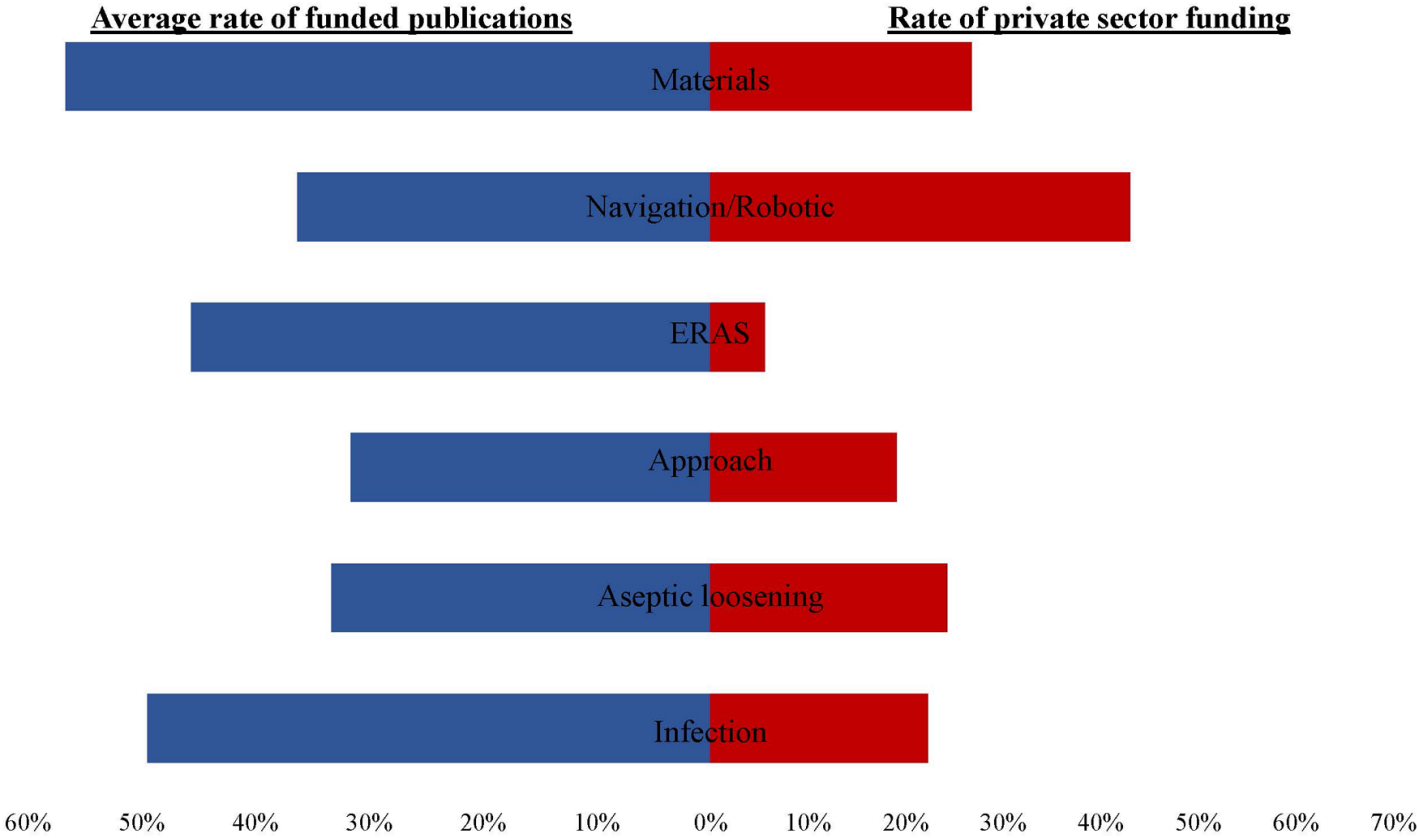
Graphic representation of the funding. The blue graph on the left shows the average rate (in percent) of publications that received funding. the red graph on the right shows the rate of funding from the private sector (in percent).

In this context, the private sector provided varying proportions of funding. Publications on the topic of navigation/robotic were by far the most frequently funded by the private sector. Publications on the topic of ERAS received below-average private-sector funding (number of private-sector funding: infection: 22.29%; aseptic loosening: 24.29%; approach: 19.11%; ERAS 6.18%; navigation/robotic: 43.02%; materials: 26.8%) (Figure 2).

### Countries

Except for ERAS, where authors from Denmark published the most, authors from the United States published the most in all topics. The distribution of publications of the leading 10 countries can be seen in Table 2.

**Table 2:** Distribution of publications by respective leading 15 countries on arthroplasty research.

Infection		Aseptic loosening		Approach		ERAS		Navigation/robotic		Materials	
USA	1,750	USA	465	USA	464	Denmark	107	USA	380	USA	1,397
Germany	458	England	160	Germany	113	USA	59	Germany	182	England	666
England	429	Germany	124	China	113	England	53	Japan	126	Germany	350
China	303	France	66	England	90	China	44	England	119	Japan	311
France	277	Switzerland	57	Japan	89	Netherlands	23	Australia	96	South Korea	213
Spain	258	Sweden	53	Switzerland	88	Germany	17	France	91	Canada	196
Switzerland	211	Japan	44	France	83	Sweden	11	South Korea	89	Italy	196
Italy	203	Finland	40	Canada	71	Australia	10	China	78	China	194
Canada	135	Italy	38	Italy	52	Canada	10	Italy	65	France	174
Netherlands	131	China	37	Australia	43	France	7	Canada	61	Switzerland	135
Sweden	123	Canada	33	Netherlands	43	Ireland	7	Switzerland	60	Australia	125
Taiwan	105	Australia	31	Austria	31	Italy	7	India	48	Netherlands	114
Australia	97	South Korea	27	Spain	28	Belgium	6	Scotland	41	Sweden	99
Other	414	Other	98	Other	114	Other	29	Other	4	Other	228

### Citations

In absolute terms, publications on the topic of infection were cited most frequently. In relation to the number of publications, the topic of aseptic loosening received the most citations (infection: 116,034 citations, 23.71 citations/publication; aseptic loosening: 34,332 citations, 26.97 citations/publication; approach: 22,955 citations, 16.14 citations/publication; ERAS: 7,785 citations, 19.96 citations/publication; navigation/robotic: 26,711 citations, 18.55 citations/publication; materials: 107,641 citations, 24.47 citations/publication). Thus, despite low publication performance, aseptic loosening showed a higher h-index than the topics approach and navigation/robotic (h-index: infection:140; aseptic loosening: 88; approach: 70; ERAS: 48, navigation/robotic: 72; materials: 129).

**Figure 3:**
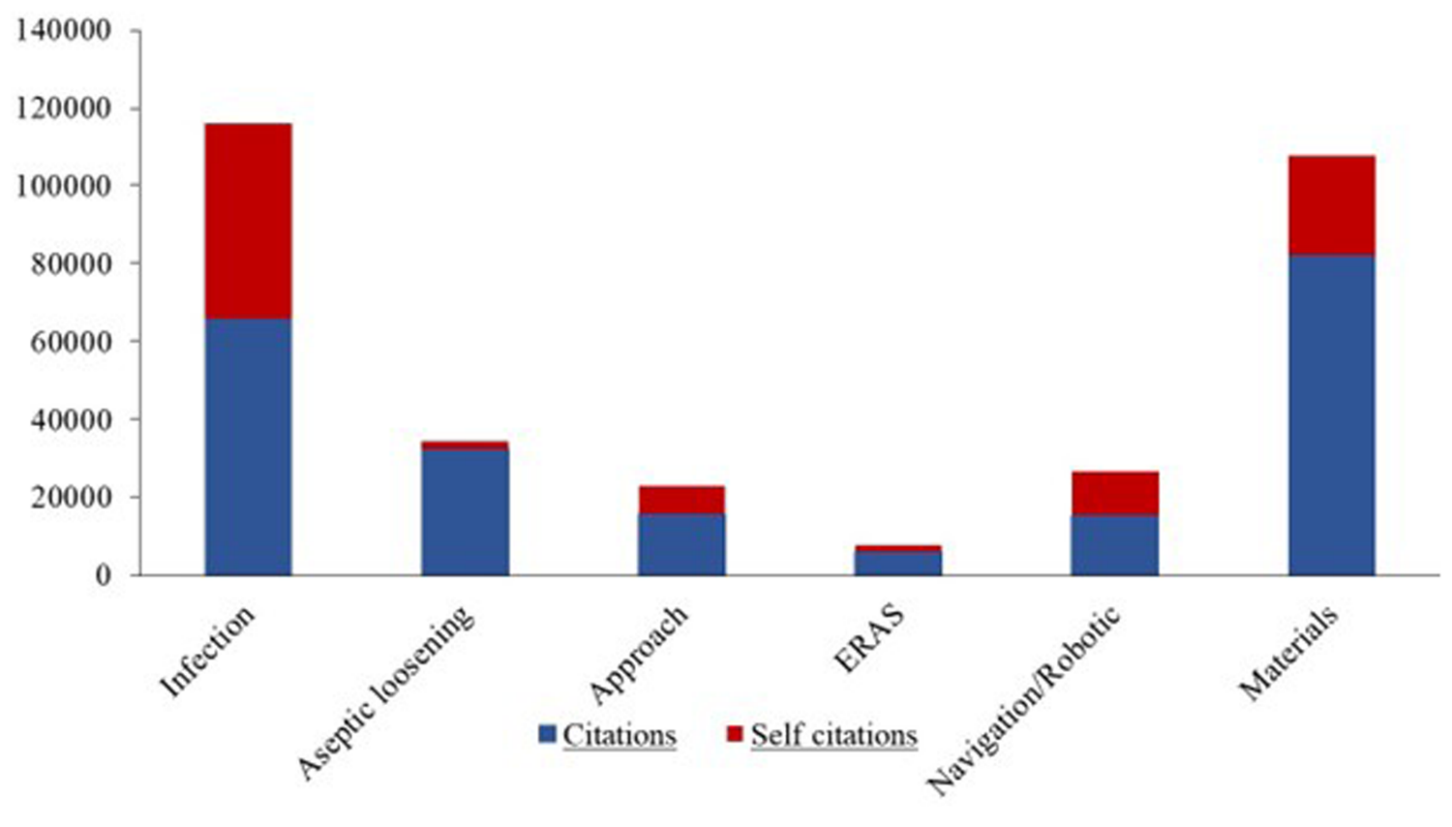
Citation analysis of subgroups. The blue fraction indicates the citations without self citations. The red share shows the self citations. The combination of the blue and the red component provides the total citations of the subgroups.

There were marked differences regarding self-citations between the various subject areas. Specifically, only 5.23% of the citations on the topic of aseptic loosening were self-citations. In the field of infection, 43.24% of all publications were self-citations (infection: 43.24%; aseptic loosening: 5.23%; approach: 31.14%; ERAS: 21.21%, navigation/robotic 42.18%; materials: 23.53%) (Figure 3).

## Discussion

In this study, we were able to show for the first time that publication patterns differ remarkably between subgroups in the research field of arthroplasty. The topics of infection and materials received a comparatively higher level of attention, whereas aseptic loosening and ERAS had fewer publications dedicated to them. Even though the number of researchers and institutes involved in each of these areas was relatively equal, the different research fields received varying degrees of funding. In the topics infection and materials, approximately every second publication received funding. For the topics aseptic loosening and approach, on the other hand, not even every third publication received funding. Despite the lower publication output in aseptic loosening, these publications received on average the highest number of citations and were published in journals with comparatively high impact factors.

For a considerable period, the research landscape has been dominated by publications focusing on new materials and types of prostheses. In the last 10 years, however, there has been a surge in the publication rate on the topic of periprosthetic infections. Most studies rank aseptic loosening as the main reason for revision surgery (30–33). However, recent study results indicate that periprosthetic infection is replacing aseptic loosening as the main reason for revision (34). The changed publication rate in the field of infection could at least be indirectly related to this finding. Thus, the scientific achievements in the long-time leading research topic materials have contributed to a longer prosthesis durability and consequently have relatively increased the relevance of the periprosthetic infections. However, it remains unclear why the most important reason for an exchange procedure, aseptic loosening (30–33), remained drastically underrepresented in the research landscape.

In general, all authors strive to publish as many papers as possible in journals with the highest possible impact factors. The number of publications, the achieved citations and h-index increase the scientific reputation of the individual and ultimately determine academic success (35–37). Self-citation can lead to an artificial increase in the h-index that does not fully correspond to the truth and should therefore be avoided wherever possible (38).

As defined in Journal Citation Reports, a self-citation does not refer to an author citing their own work, but rather to a citation of a work from the same journal in which the author's research is published. Therefore, the high self-citation rate may be attributed to a more concentrated distribution of publications across fewer journals, leading to a higher rate of citations within a given journal. However, whether this is the sole reason for the significant discrepancy requires further investigation through self-citation-centered bibliometric studies. The relatively new research fields of ERAS and navigation/robotic have become increasingly relevant in recent years.Navigation/robotic, supported by the highest funding rate from the private sector, has recorded a distinct increase in publications. In addition to funding, this can certainly be explained by advances in computer technology and the general progress of digitization.

The clinical and scientific landscape in the field of joint replacement is currently characterized by increasing technologization, as demonstrated in this study. The new digital possibilities are intended to increase intraoperative precision and improve outcomes (39). The increasing willingness to publish in this area can also be directly attributed to the extensive financial support available, which also applies to the field of infections. With regard to financial interests, particular attention should also be paid to the costs associated with periprosthetic joint infections and the private sector's interests in promoting navigation/robotics. Regardless, clinically highly relevant areas such as aseptic loosening should also receive increased financial support. This could overcome the shortcomings in publication output and improve the longevity of implanted prostheses in the future.”

### Limitations

Like all bibliometric studies, this study is subject to some limitations. Unspecific search terms result in off-topic publications, while over-specific combinations can lead to the exclusion of certain relevant publications. Even though the Web of Science databases are among the most comprehensive databases, not all publications are represented and there are deficits especially with regard to non-English publications (24). The use of data from several databases in one study would be advantageous but requires the support of external software programs, which in their turn are subject to their own limitations (40–47). Affiliations to nations are determined by the nationality of the first author, possibly reducing multicentre studies to this one nation. Strengths of this study include a first scientific comparative study of the research situation in the field of arthroplasty, as well as insights in the differences in the scientific interest between the various topic areas.

## Conclusion

In this bibliometric subgroup analysis, the primary scientific outputs focused on septic complications and materials research in the field of arthroplasty, which is also reflected in a higher rate of financial funding in these topics. Even if publications on aseptic loosening were less frequently published and financially supported, the publications were cited more frequently on average. Even though a bibliometric work can never make absolute statements on the contents due to the underlying methodology, it can be concluded that the clinically highly relevant research field of aseptic loosening should receive increased financial support. This will promote the improvement of patient care in the long term.

## Data Availability

The original contributions presented in the study are included in the article, further inquiries can be directed to the corresponding author.
